# Potential Role of CD99 Signaling Pathway in Schwann Cell Dysfunction in Diabetic Foot Ulcers Based on Single-Cell Transcriptome Analysis

**DOI:** 10.1155/jdr/9935400

**Published:** 2025-05-18

**Authors:** Yannan Zhou, Yaxin Zhou, Haohan Chen, Li Zhang, Siwei Bi

**Affiliations:** ^1^Department of Burn and Plastic Surgery, West China Hospital, Sichuan University, Chengdu, Sichuan Province, China; ^2^Department of Medical Ultrasound, West China Hospital, Sichuan University, Chengdu, Sichuan Province, China; ^3^Department of Cardiovascular Surgery, West China Hospital, Sichuan University, Chengdu, Sichuan Province, China

**Keywords:** CD99, diabetic foot ulcer, Schwann cell, single-cell transcriptome

## Abstract

**Background:** Schwann cell (SC) dysfunction contributes to the delayed healing of diabetic foot ulcers (DFUs). However, the underlying molecular mechanism regarding the unregulated SC function is poorly understood. Thus, we examined the single-cell transcriptome data from different DFU states focusing on SC characteristics.

**Methods:** The single-cell RNA sequencing (scRNA-seq) data of DFU was obtained from the Gene Expression Omnibus (GEO) database, covering foot skin samples from nondiabetic patients, diabetic patients without DFU, DFU healers, and DFU nonhealers. After scRNA-seq data processing, downscaling, and cell cluster identification, cell communication analysis was performed by the CellChat package. Furthermore, we subclustered SC populations and ran the trajectory inference and pseudotime analysis to investigate the dynamic changes in SC. Finally, the significant pathways were validated with a *db/db* mouse wound model.

**Results:** scRNA-seq analysis revealed different SC percentages and gene markers across the DFU groups. We identified that the CD99 signaling pathway was upregulated in the DFU nonhealer group. In the *db/db* mouse wound model, we observed that CD99 was highly expressed in the demyelinated area of the peripheral nerve fibers.

**Conclusion:** Our study elucidated that the CD99 pathway activation may play a crucial role in SC dysfunction of DFU, providing insights into the peripheral glia regulation mechanism and potential therapeutic target of DFU.

## 1. Introduction

Diabetes mellitus is now a leading and rapidly increasing cause of global disease and disability burden [1, 2]. Diabetic foot ulcer (DFU) is one of the diabetes-related lower extremity foot complications [3], and approximately 34% of patients with diabetes develop DFU in their lifetime [4]. People with DFU are at high risk of developing infections and progressive gangrene, requiring hospitalization and amputation eventually in severe cases [5, 6]. It has been estimated that the 5-year mortality for DFU and DFU with major amputation was 30% and 56%, respectively [7]. DFU has also been reported to portend a 2.45 times higher rate of all-cause mortality compared with diabetes alone [8].

Peripheral neuropathy takes an important part in DFU development, which encompasses sensory, motor, and autonomic neuropathy [4]. Schwann cell (SC) is the main glial cell of the peripheral nerve and is responsible for the formation of peripheral nerve myelin. SC is important in maintaining peripheral nerve function and repair after peripheral nerve injury and is closely related to the axons for material exchange [9]. Apart from the repair capacity of the peripheral nerve system, SC has been proven to promote wound healing. Skin injury activates the dedifferentiation of SC that proliferates and disseminates into the wound bed to promote wound healing [10]. Increasing evidence indicates that the repair performance of SC was achieved through regulating nonneural tissue stem cell populations via paracrine signaling [11]. Particularly, “repair” SC induces myofibroblast differentiation from fibroblasts via TGF-*β* signaling, an essential basis of extracellular matrix (ECM) remodeling in the early stage of the normal wound healing process [12, 13]. Nonetheless, the diabetic environment can induce SC's apoptosis mainly mediated by oxidative stress, inflammation response, endoplasmic reticulum stress, and autophagy [9]. This will lead to nerve fiber demyelination and a prolonged healing process. Studies have shown that compared with normal skin, SC in diabetic skin diminished and showed functional deficiencies in cell dedifferentiation, re-entry into the cell cycle, cell wound bed migration, and reduction in paracrine TGF-*β* signaling [12, 14].

Regarding the complicated clinical outcomes of DFU and the comprehensive profiles of SC, the mechanism by which SC affects DFU and the subsequent wound healing process is not entirely elucidated. Single-cell RNA sequencing (scRNA-seq) is a recently emerging and promising tool to identify distinct cell types in specific tissues. With the development of the scRNA-seq technique, more detailed information about cellular alterations and communications in DFU has been revealed [15, 16]. In 2022, Theocharidis et al. conducted a scRNA-seq analysis and provided a well-conserved dataset concerning DFU samples [17]. Therefore, to shed light on the shifting characteristics of SC in DFU, we harnessed the dataset focusing on the SC and identified the different cell signaling pathways. Additionally, the in vivo DFU model was applied to validate the findings.

## 2. Method

### 2.1. Data Acquisition

The scRNA-seq data of DFU were downloaded from the Gene Expression Omnibus (GEO) database (https://www.ncbi.nlm.nih.gov/geo/), accession number GSE165816, comprising 33 foot skin samples: 11 control samples from nondiabetic patients, 8 samples from diabetic patients without DFU, 9 samples from DFU healers, and 5 samples from DFU nonhealers. Foot skin samples of nondiabetic and diabetic patients without DFU groups were collected from patients who underwent foot surgery for various reasons. Wound tissue samples of the DFU healer and DFU nonhealer groups were collected from diabetic patients with DFU at 12 weeks after resection surgery of the ulcer.

### 2.2. Data Processing

#### 2.2.1. Quality Control and Data Integration

Quality control was performed with the Seurat R package (Version 4.4.0), by filtering low-quality and dying cells. Following the tutorial (https://www.singlecellcourse.org), we eliminated cells where more than 5% of total counts were attributed to either mitochondrial protein genes, ribosomal genes, or hemoglobin proteins via the PercentageFeatureSet function. The DoubleFinder R package was used to remove putative doublets [18]. Then, SCTransform was applied to filtered gene expression matrices of each sample. The SelectIntegrationFeatures function, with the number of variable genes set to 3000, and the PrepSCTIntegration and FindIntegrationAnchors functions were used to integrate the dataset through the IntegrateData function. Eventually, the integrated data was included in subsequent analysis.

#### 2.2.2. Cell Clustering and Annotation

Principal component analysis (PCA) was conducted in the four DFU groups for dimensionality reduction. The Top 30 principal components (PCs) were then extracted and clustered using the Louvain algorithm, with a resolution parameter of 0.4. Uniform manifold approximation and projection (UMAP) was used to visualize the result. The main clusters were annotated according to known cell markers using the FindAllMarkers function.

#### 2.2.3. Cell–Cell Communication

The cell–cell interactions were applied for the four DFU groups using the CellChat algorithm (https://github.com/sqjin/CellChat (Version 2.0.0)) [19]. We put the Seurat data into CellChat for processing, adhering to the official procedure. First, the key incoming and outgoing signals were predicted for specific cell types based on the ligand–receptor database to identify the dominant target (signal receiver) and sender (signal source) cells. The CellChat algorithm was then performed to calculate the possible interactions and pathways through the “computeCommunProb” and “computeCommunProbPathway.” Intercellular pathways were divided into two types, incoming or outgoing patterns, revealing how target (signal receivers) or sender (signal sources) cells coordinate with other cells, respectively. Moreover, we achieved ligand–receptor pairs' annotation to signaling pathways using a CellChatDB database or custom annotations and probability aggregation. Then, the aggregated probabilities were normalized to account for differences in the number of ligand–receptor pairs per pathway. The pathway-level communication probabilities are stored in the netP slot of the CellChat object for further analysis. Additionally, we applied the “filterCommunication” to exclude interactions with less than 10 cells in each group of cells.

#### 2.2.4. Pseudotime Analysis of SC

We first separated the SC population from all DFU cells in each sample using the subset function and then performed the trajectory inference and pseudotime analysis using the Monocle3 package (https://cole-trapnell-lab.github.io/monocle3) [20]. A principal graph was created using the learn graph function, followed by assigning pseudotime values to cells through order_cells and a manually chosen pseudotime start or “root” position. Genes differentially expressed along the estimated trajectories were identified using the graph_test function to track changes over pseudotime.

#### 2.2.5. Gene Analysis of SC

Following the Seurat tutorial, we clustered the SC further. Differentially expressed genes (DEGs) were calculated with “FindAllMarkers” function to determine the different expression profiles in clusters. The minimal log fold change was set to 0.5 and only positive markers identified (*p* values < 0.05). The DEG analysis was conducted using the Wilcoxon rank-sum test, considering only genes detected in over 50% of cells in either population.

### 2.3. Animal Model Establishment and Immunofluorescence Staining

Seven-week-old male wild-type C57BL/6 and *db/db* mice were bought from Dashuo company (Chengdu, China). Mice were kept in groups of four in cages under particular pathogen-free settings. All animal procedures were approved by the Animal Ethic Review Committees of the West China Hospital (Approval No. 20240307071), following the Institutional Guidelines for the Care and Use of Animals. Mice were given full-thickness (1 cm) incisions on their backs. Briefly, isoflurane was used to anaesthetize the mice. The back skin was then shaved. Wounds were created by 10-mm punch biopsy needles. The wounded tissues of mice on Day 7 were collected. The indirect immunofluorescence assay was applied. The antibodies of CD99 and MBP were used to evaluate CD99 expression and peripheral nerve distribution. Negative control by only fluorescent secondary antibody was implemented to account for antibody specificity. Collected skin tissues were sliced, deparaffinized, rehydrated, and processed with sodium citrate solution. Then, the samples were incubated with the designated antibodies (CD99: ER1803-81, HUABIO, Woburn Massachusetts; MBP: sc-271524, Santa Cruz Biotechnology, Dallas, Texas) overnight at 4°C before staining. ImageJ software quantified the staining intensity (National Institute of Health, Bethesda, Maryland).

### 2.4. Statistical Analysis

The continuous variables were represented by means ± standard error if normally distributed and median (quartiles) if nonnormally distributed. The categorical variables were represented as frequencies and percentages. The Student's *t*-test, one-way analysis of variance (ANOVA) test, or Mann–Whitney *U*-test was used for comparisons between groups. *p* < 0.05 was considered statistically significant. Statistical analysis was performed using R studio (Version 4.3.1).

## 3. Results

### 3.1. Identification and Gene Features of DFU Cells

**Figure 1:**
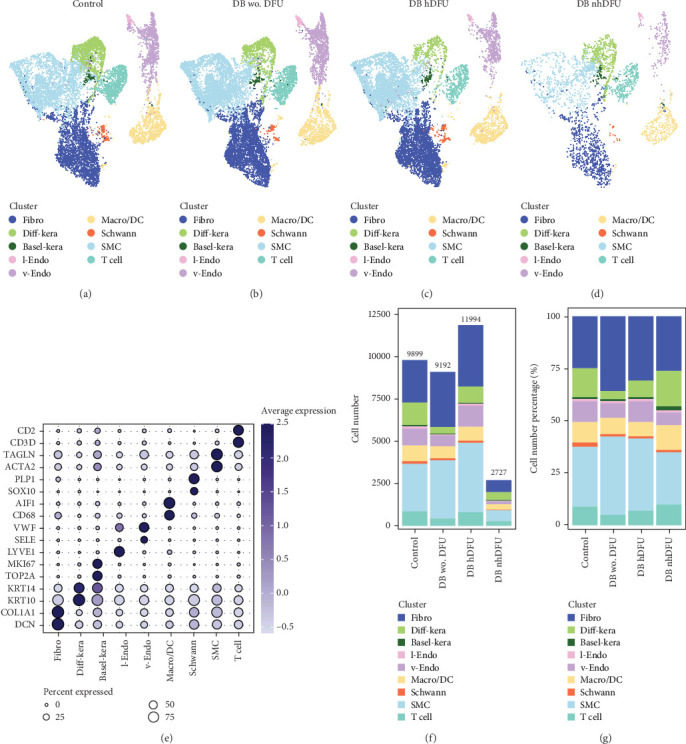
Single-cell analysis of diabetic foot ulcer (DFU) scRNA-seq data. Uniform manifold approximation and projection (UMAP) plot for (a) control, (b) diabetic (DB) patients without DFU, (c) DFU healers, and (d) DFU nonhealers after aggregation of all datasets. Cluster identifications of fibroblasts, smooth muscle cells, basal and differentiated keratinocytes, vascular- and lymphatic-endothelial cells, and macrophage/dendritic cells. (e) The expression levels of well-known marker genes to classify cell types. The (f) absolute number and (g) percentage of each cell type in DFU scRNA-seq data.

scRNA-seq libraries covering the different statuses of DFU-related skin samples were merged, respectively, to build a cellular atlas in foot skin tissue of nondiabetic patients, diabetic patients without DFU, DFU healers, and DFU nonhealers (Figures 1, 1, 1, and 1). Given the relatively smaller number of samples in the DFU nonhealer group (*n* = 5), there are fewer cells in this group compared with other groups. Based on the expression patterns of well-known marker genes, nine main cell types were determined (Figure 1). Fibroblasts and smooth muscle cells (SMCs) are the two largest cell clusters. The fibroblasts expressed genes linked to fibrosis and ECM accumulation and were marked by elevated DCN and Col1a1 expressions. The other cells were also marked, such as ATAC2 for SMCs, TOP2A for differentiated keratinocyte (diff-kera), KRT14 and KRT10 for basal keratinocyte (basal-kera), LYVE1 for lymphatic endothelial cells (l-endo), VWF for vascular endothelial cells (v-endo), CD68 for macrophage/dendritic cells (Macro/DC), CD2 for T cells, and SOX10 and PLP1 for SCs as previously established. The composition of cells varied substantially between the four DFU groups (Figure 1). Both diabetic without DFU and DFU healer groups showed higher proportions of fibroblasts than the control group, while such proportions were comparable between the DFU nonhealer and control groups. The relative abundance of differentiated keratinocytes was greater in the DFU nonhealer group than in the other two diabetic groups. In addition, there was a significantly lower percentage of SC detected in the DFU healer (mean difference = 0.84, 95% CI: 0.15–1.53, *p* < 0.05) and DFU nonhealer (mean difference = 0.96, 95% CI: 0.14–1.79, *p* < 0.05) groups than the control group. The diabetic patients without DFU were also observed with lower SC proportion, but the difference is not significant (mean difference = 0.51, 95% CI: −0.18–1.20, *p* > 0.05) (Figure S3).

### 3.2. Characteristics of SC in DFU Skin

**Figure 2:**
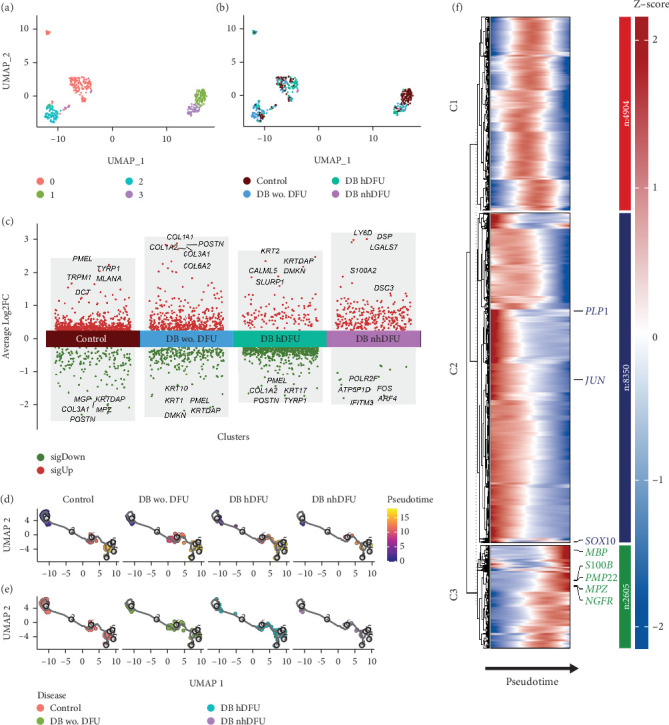
UMAP plot for Schwann cell (SC) colored by (a) Seurat cluster identity and (b) diabetic foot ulcer (DFU) group. (c) Significantly differentially expressed genes in the four DFU groups. (d) Pseudotime analyses delineate putative SC trajectories with cells color-coded by pseudotime and (e) DFU groups. (f) Heatmap displaying the dynamically changing genes of SC arranged based on the pseudotime.

Previous evidence has indicated the role of SC dedifferentiation in wound healing promotion. Thus, we proposed the differentiation level could be dissimilar among the DFU groups and conducted a comprehensive analysis of detailed SC characteristics between different DFU statuses focusing on the dedifferentiation markers. We first subclustered the SC scRNA-seq data as shown in the UMAP analysis (Figure 2). The clustering results showed that the SC in the control group can be different from those in other groups as distinguished by the unsupervised clustering. Nonetheless, SCs in the DFU nonhealer group are difficult to distinguish given the relatively lower cell number and the scattering position. Significant DEGs were analyzed across DFU-related groups (Figure 2). Generally, more genes were upregulated in the control and DFU nonhealers while downregulated in the DFU healers. Intriguingly, the MPZ gene was downregulated in the control group. The MPZ gene exhibits specific expression in the SC of the peripheral nervous system and encodes a Type I transmembrane glycoprotein, which serves as a major structural component of the peripheral myelin sheath. The encoded protein is indispensable in both the formation and stabilization of the multilamellar structure within the compact myelin. Moreover, with the monocle3 package, the pseudotime trajectories of SC revealed similar cell proportions in the pseudotime line to some extent across all the groups (Figure 2). As shown in Figure 2, to further investigate the SC trajectory and the dedifferentiation status, we profiled several important regulators and markers of SC: quiescent SCmarker (S100B), peripheral glial lineage marker (SOX10), myelination markers (MBP, PLP1, MPZ, and PMP22), and dedifferentiation markers (JUN and NGFR/p75). Pseudotime trajectory recaptured the differentiation process of SC, where the expression of quiescent and myelination markers was increased with the pseudotime. Intriguingly, the JUN and NGFR showed different expression trends, and a solid conclusion about the dedifferentiated status along the pseudotime could not be drawn. Although the pseudotime trajectories results resonate with the UMAP results in Figure 2, indicating that the difference of SC in these four groups was not distinguishable, one possible reason for the chronic healing process could be due to the scarce number of SC shown in the DFU nonhealer group.

### 3.3. Discriminated Signaling Pathways in SC in DFU Skin

**Figure 3:**
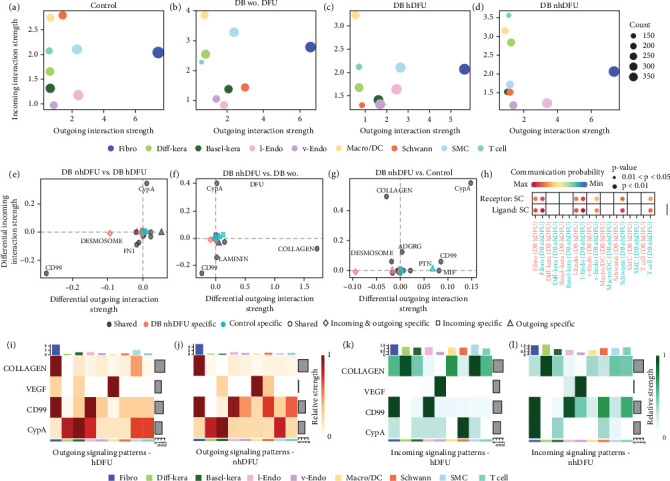
Incoming and outgoing interaction strength scatter plots in different cell types of (a) control, (b) diabetic (DB) patients without diabetic foot ulcer (DFU), (c) DFU healers, and (d) DFU nonhealers. Differential signaling pathways' interaction strength analysis of SC between DFU nonhealers versus (e) DFU healers, (f) DFU nonhealers versus DB patients without DFU, and (g)DFU nonhealers versus control. Positive values indicate an increase in the second dataset while negative values signify an increase in the first dataset. Cell chat ligand and receptor analysis for CD99 signaling pathway communication probability, (h) SC: Schwann cell. (i–l) Heatmap showing the incoming and outgoing signaling strength in selected pathways in DFU non-healers and DFU healers.

To further identify the signaling of SC in the DFU wound healing process and the reason for scarce SC in DFU nonhealers, we applied CellChat to examine SC's cell signaling profile features in different groups. Overall, DFU nonhealers and diabetic patients without DFU groups showed comparable interaction status, with a lower number but higher total interaction strength than the other two groups (Figure S1). As shown in Figures 3, 3, 3, and 3, the *x*-axis and *y*-axis of the scatter plot showed the total incoming or outgoing communication strength referred to each cell group, respectively. The size of dot is proportional to the number of inferred links. The incoming or outgoing communication strength reveals how active the cell population is as a target cell or a sender cell. Cells with high outgoing strength and low incoming strength may act as an upstream regulator in a certain signal pathway, while those with low outgoing strength and high incoming strength are more likely to be responders. Mediator cells tend to express similar incoming and outgoing strength. The fibroblasts sent the most abundant signals in all four groups, which underlies their significant role in the DFU healing process. On the other hand, the incoming signal strength of the SMC/DC cluster ranked first or second in all four groups. The signal that SC received was particularly remarkable in the control group and trivial in the DFU healer group. The incoming and outgoing signal strength in SC of the DFU nonhealers was relatively weak compared with the other groups. We also visualized the difference in outgoing and incoming signaling associated with SC cluster between the control, diabetic without DFU, or DFU healer group and DFU nonhealer group (Figure 3, 3, and 3). The SC in the DFU nonhealer group showed a decreased incoming CypA signaling pathway in all three comparisons. However, when comparing DFU nonhealers versus DFU healers, the CD99 signaling pathway was increased in both the incoming and outgoing directions. Furthermore, the ligand–receptor (L-R) analysis showed specific CD99 signal interactions in SC, as both ligand and receptor, with vascular endothelial, SC, and T cells in the DFU nonhealer group, which was not present in DFU healers (Figure 3). The chord plot for the CD99 and CypA signaling results were illustrated in Figure S2. Similarly, the CD99 pathway was enriched between vascular endothelial cells and SC, and between T cells and SC in the DFU nonhealer group compared with the DFU healer group. The CypA pathway was reduced from vascular endothelial cell to SC, and from T cell to SC in the DFU nonhealer group compared with the DFU healer group. For a more specific comparison of SC in DFU nonhealers versus DFU healers, we selected four pathways: collagen, vascular endothelial growth factor (VEGF), CD99, and CypA for the heat map illustration of incoming and outgoing signals in all cell clusters (Figures 3, 3, 3, and 3). The color represents the associated signaling strength of a signaling pathway across cell groups. The bar plot at the top shows the cumulative signaling strength of a group of cells by summarizing all displayed signaling pathways in the heat map. Furthermore, the grey bar plot on the right showcases the overall signaling strength of a signaling pathway by aggregating data from all cell groups presented in the heat map. SMC and SC showed significantly different profiles in the collagen pathway and CD99 pathway, respectively, when comparing the DFU nonhealers versus DFU healers in both incoming and outgoing signals.

### 3.4. Elevated Expression of CD99 in SC of Diabetic Wound

**Figure 4:**
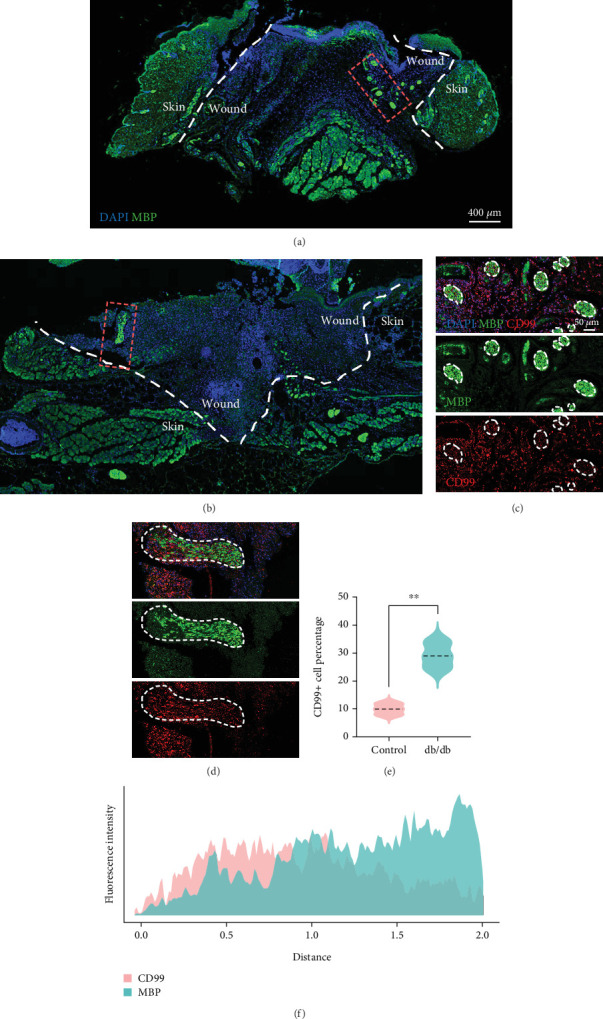
Validation of CD99 expression in Schwann cell (SC) of diabetic (DB) mice. Representative fluorescent images of MBP and CD99 staining of the skin wound collected from (a) control and (b) *db/db* mice. Scale bar = 400*  μ*m. (c, d) Immunostaining of MBP and CD99 in the nerve region (white circle) in the wound from control and *db/db* mice. Scale bar = 50*  μ*m. (e) Quantification of CD99^+^ cell numbers in control and *db/db* mice. (f) Fluorescence distribution of CD99 and MBP in the nerve region of the wound from *db/db* mice. ⁣^∗^*p* < 0.05, ⁣^∗∗^*p* < 0.01, and ⁣^∗∗∗^*p* < 0.001.

To confirm the CD99 expression of SC in diabetic wound skin, we collected the wound samples from the control and *db/db* mice and performed immunofluorescent staining based on MBP and CD99. Peripheral nerves were marked by MBP. The negative control staining of the fluorescent secondary antibody confirmed the specificity of the antibody (Figure S4). The immunostaining results demonstrated that significantly fewer myelinated nerve fibers were observed in *db/db* mice compared with control mice, indicating the demyelination and damage of peripheral nerves in diabetic wound skin (Figure 4). As depicted in Figure 4, the cell number percentage with CD99 fluorescence in the *db/db* mice was significantly higher than that in the control mice (Figure 4). Specifically, demyelination could be observed in the *db/db* mice as the shrinkage of fluorescence of MBP in the peripheral nerve area (left side), where CD99 fluorescence intensity was higher (Figure 4). This result suggested the potential role of CD99 in peripheral demyelination and SC dysfunction.

## 4. Discussion

Although numerous studies have explored the pathophysiology of DFU, the underlying cellular and molecular mechanism remains inadequate, posing challenges in the management and treatment of DFU. Advancements in cell informatics technology have revealed an increasing number of molecular signaling pathways in the healing process of DFU, primarily focusing on inflammation response and immune reaction [21]. Notably, the peripheral nerve system dysfunction proves to be pivotal in the wound healing process of DFU. Previous evidence has suggested that diminished repair response by SC is attributed to the delayed healing of DFU. However, evidence of the specific mechanism is relatively short. To gain deeper insights into nerve regulation in DFU, we generated a single-cell transcriptome analysis examining SC's shifting profiles. The results showed that the wound tissue from DFU healers and DFU nonhealers presented distinct SC states. Moreover, our study identified CD99 signaling pathway significantly upregulated in the DFU nonhealer group compared with the DFU healer group. We also observed that CD99 exhibited high expression levels within the demyelinated area of peripheral nerves in DFU skin samples, indicating its potential role in SC reduction and dysfunction.

SC's death in the DFU is intriguing. As aforementioned, previous evidence believed that several pathological processes could trigger SC apoptosis under hyperglycemia circumstances [9]. Significantly, our results suggest the potential role of CD99-mediated cell death in DFU. CD99 is a transmembrane protein involved in many fundamental biological processes, such as apoptosis, cell adhesion, and migration. It also participates in the inflammation processes, immune responses, and neural precursor cell differentiation [22]. Under normal circumstances, CD99 is highly expressed in immature lymphocytes and granulocytes, while displaying low expression levels in neural cells. The activation of CD99-mediated signaling is believed to be initiated by homophilic contacts between CD99 molecules on contacting cells. Thus, the physiological function of CD99 is mainly defined by the expression levels of CD99 [23]. The current exploration of CD99 primarily lies in its implications for cell death in tumors [24]. Forms of regulated cell death mainly included apoptosis and nonapoptosis pathways (necroptosis, pyroptosis, ferroptosis, etc.). Apoptosis signals are transduced by BCL-2 proteins and caspases, culminating in the breakdown of nuclear membrane, the cleavage of intracellular proteins, membrane blebbing, and the breakdown of genomic DNA [25, 26]. Non-apoptosis process is characterized by the loss of cellular integrity and intracellular contents destabilization, but the signal pathways vary across different pathological situations [25]. Necroptosis is mainly triggered by ligand and death receptor pairings such as FAS ligand with FAS and tumor necrosis factor (TNF) with TNF receptor 1. Gasdermin pores in the plasma membrane and phospholipid peroxides in cell membranes induce pyroptosis and ferroptosis, respectively [27]. In tumors, CD99 interaction induces cell death via an unconventional, caspase-independent apoptotic pathway or a nonapoptotic pathway resembling methuosis [22]. Methuosis is differentiated from other nonapoptotic pathways by large fluid-filled cytoplasmic vacuole accumulation. The vacuoles form from macropinosomes and can compromise cell viability [28]. Recently, Manara et al. clarified that the engagement of CD99 and antibody against CD99 (0662mAb) would elicit caveolin-1-dependent endocytosis with the downstream response between the IGF-1R and RAS/Rac1, thus initiating processes akin to methuosis in Ewing sarcoma cells [29]. Nevertheless, it is interesting to note that IGF-1R is the receptor to IGF-1, a pluripotent growth factor expressed in SC. The expressions of IGF-1 and IGF-1R were dysregulated in diabetic patients and considered to be associated with the defects of peripheral nerve myelination in diabetic neuropathy [30, 31]. Accordingly, the significance of methuosis-like process may warrant more attention in future research regarding mechanisms underlying CD99-induced SC cell death in DFU.

The flexible differentiation state of SC plays a crucial role in skin injury repair by promoting nerve regeneration and dermal wound healing. Mature SC will undergo transcriptional reprogramming after peripheral nerve injury, including dedifferentiation into immature cell conditions and acquisition of repair-specific abilities [32]. Dedifferentiated SC guided nerve regeneration via upregulation of growth factors, elevation of natural immune cytokines, myelin clearance by activation of myelin autophagy in SC, and recruitment of macrophages, as well as facilitating the formation of regeneration tracks [33, 34]. On the other hand, dedifferentiated SC can emanate from the disrupted nerve and support the transformation of fibroblasts into myofibroblasts, promoting wound contraction and closure as well as reepithelization [10]. Direder et al. investigated the transcription profile of SC in keloid formation and identified the contribution of a nonclassical repair-like SC to the overproduction of the ECM [35]. Moreover, Ou et al. demonstrated that the differentiated SC can promote the migration of fibroblasts and keratinocytes by secreting TGF-*β*3 [14]. In our study, we compared the detailed SC characteristics between different DFU statuses focusing on the dedifferentiation markers and found that the subclusters of SC were different among groups. However, this result was limited by the relatively scarce number of SC in the DFU nonhealer group. Given the dural functions of SC on neural regeneration and dermal repair, the differentiation signature of SC may be complicated in DFU skin and warrants more research.

Cell communication analysis showed that when comparing the DFU nonhealer group with the DFU healer group, the CD99 signaling pathway was enhanced between vascular endothelial cell and SC, as well as between T cell and SC. The interaction between SC and vascular endothelial cell has been reported to be involved in the regeneration process of injured peripheral nerves. Endothelial cells remove degenerative and necrotic materials from the lesion site and supply the essential nutrients during the early stages of peripheral nerve regeneration [33, 36]. Moreover, endothelial cells are crucial for guiding SC migration and specific reconnection of axons to respective organs [37]. According to Huang et al., endothelial cell-derived exosomes can enhance and sustain the SC repair-related phenotypes promoting remyelination, axonal regeneration, and angiogenesis [38]. At the same time, SC was found to promote endothelial migration in the co-culture model of human umbilical vein endothelial cells and rat SC [39]. Immune response is also significant in nerve repair and diabetic wound healing processes [40–42]. SC has been demonstrated as immune-competent cells that contribute to inflammatory and hereditary neuropathies [43]. Repair-related SC possesses features of antigen-presenting cells and is capable of mediating T cell-dependent immunity [44]. SC can interact with T cells by expressing major histocompatibility Complex Class II receptors and cosignaling molecules. Hartlehnert et al. demonstrated that T cell activation through MHC II expressing SC was associated with posttraumatic inflammation in diseased peripheral nerves of mice [45]. The distinguished expression of the CD99 signaling pathway between vascular endothelial cell, T cell, and SC indicated their potential interaction in the chronic healing process of DFU.

There are several limitations in our study. First, our results are based on bioinformatic analyses and lack excitement and inhibition validation experiments. Additionally, the number of DFU samples was limited in the scRNA-seq data. Larger sample sizes with animal models or human samples are required for further research. Third, the number of SC is rare in the DFU nonhealer group, which hinders detailed and comprehensive information for SC states and differentiation, as well as undermines the strength of pseudotime and cell communication analyses.

## 5. Conclusion

The study identified that the activation of the CD99 signaling pathway could be associated with SC dysfunction in DFU, resulting in demyelination and the chronic wound healing process. This finding promotes the mechanism of SC shifting signature in DFU and provides a potential target for restoring the SC in DFU and improving the DFU therapies.

## Data Availability

The data that support the findings of this study are available from the corresponding author upon reasonable request.

## Supporting Information

Supporting Information Additional supporting information can be found online in the Supporting Information section. Figure S1: Bar plot showing the number of inferred interactions and strength using CellChat. Figure S2: Chord plot showing the ligand and receptor in CD99 and CypA signaling pathways. Figure S3: The Schwann cell percentage in different DFU groups. Figure S4: The fluorescent image of negative control staining of skin wound.
