# Supplementary material for: Potential Role of CD99 Signaling Pathway in Schwann Cell Dysfunction in Diabetic Foot Ulcers Based on Single-Cell Transcriptome Analysis
Source: J Diabetes Res. 2025 May 18;2025:9935400. doi: 10.1155/jdr/9935400 (PMC12103954; doi:10.1155/jdr/9935400)
Supplement: Supporting Information — Additional supporting information can be found online in the Supporting Information section. Figure S1: Bar plot showing the number of inferred interactions and strength using CellChat. Figure S2: Chord plot showing the ligand and receptor in CD99 and CypA signaling pathways. Figure S3: The Schwann cell percentage in different DFU groups. Figure S4: The fluorescent image of negative control staining of skin wound. [file 9935400.f1.docx]

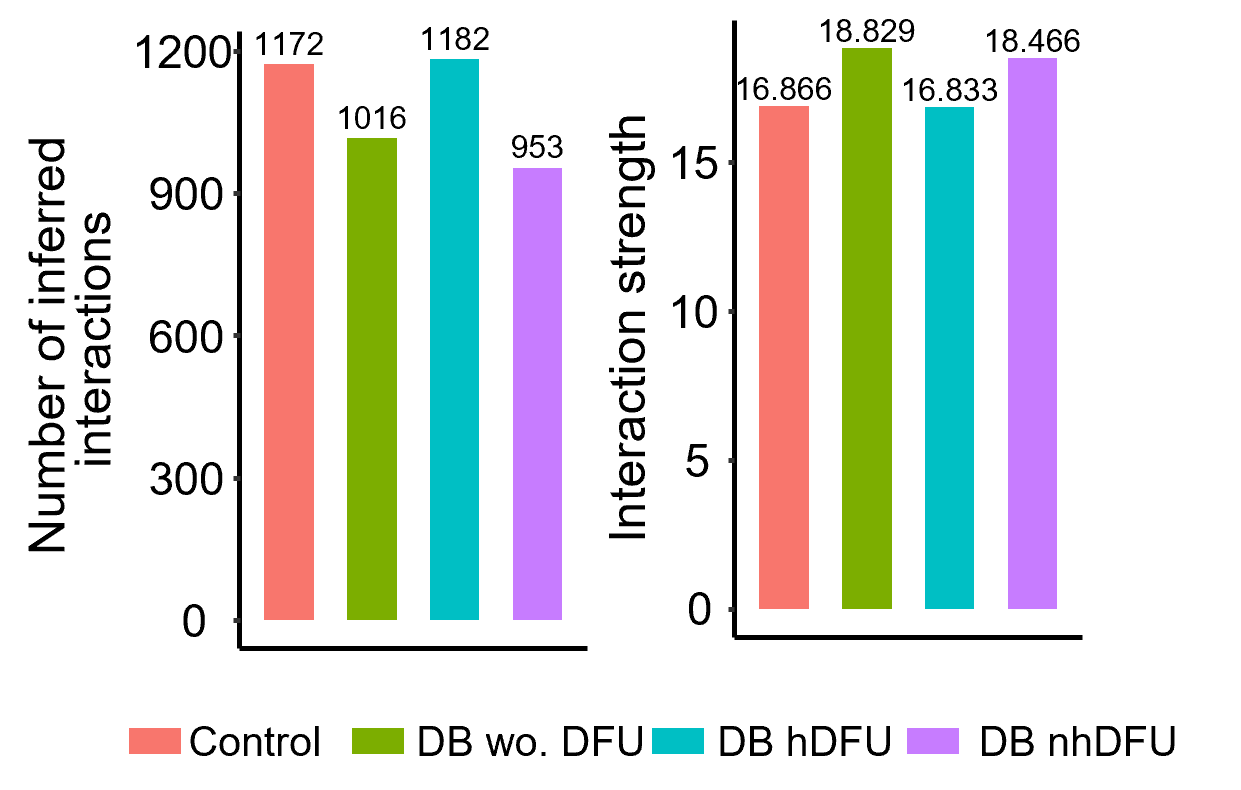
Supplementary Figure 1: Bar plot showing the number of inferred interactions and strength using CellChat.


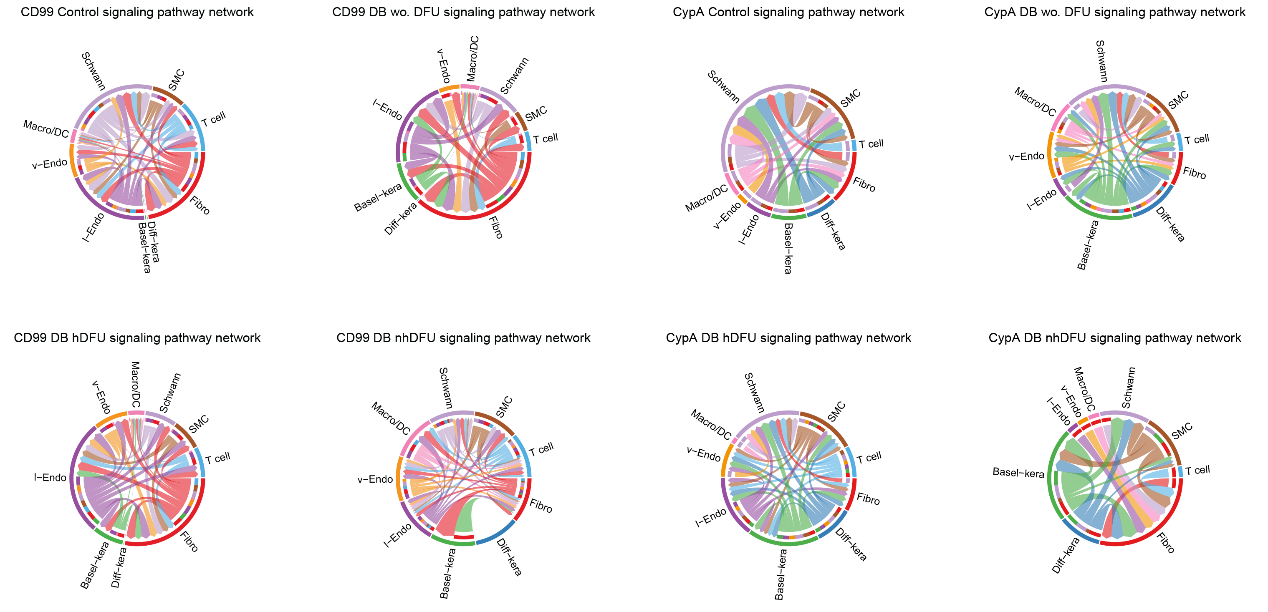


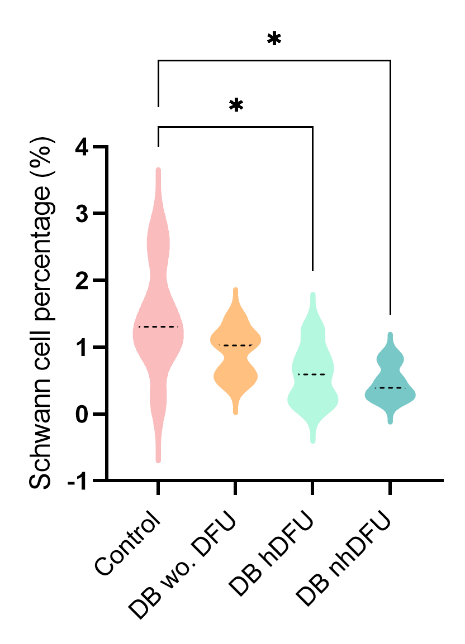
Supplementary Figure 2: Chord plot showing the ligand and receptor in CD99 and CypA signaling pathways.

Supplementary Figure 3: The Schwann cell percentage in different DFU groups.


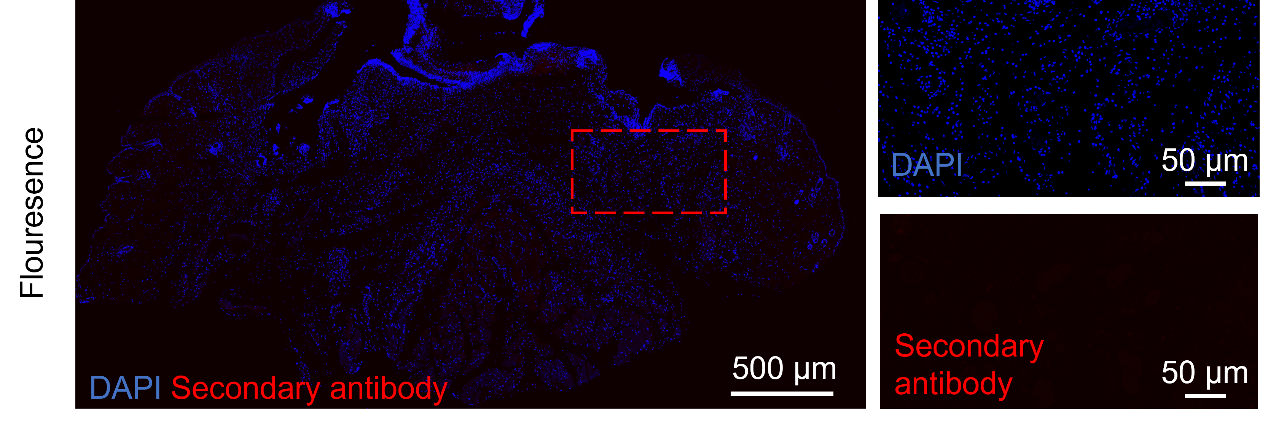


Supplementary Figure 4: The fluorescent image of negative control staining of skin wound.
